# Conformational flexibility of apolipoprotein A-I amino- and carboxy-termini is necessary for lipid binding but not cholesterol efflux

**DOI:** 10.1016/j.jlr.2022.100168

**Published:** 2022-01-17

**Authors:** Shimpi Bedi, Jamie Morris, Amy Shah, Rachel C. Hart, W. Gray Jerome, Stephen G. Aller, Chongren Tang, Tomas Vaisar, Karin E. Bornfeldt, Jere P. Segrest, Jay W. Heinecke, W. Sean Davidson

**Affiliations:** 1 Department of Pathology and Laboratory Medicine, University of Cincinnati, Cincinnati, OH, USA; 2 Department of Pediatrics, Cincinnati Children’s Hospital Medical Center, Cincinnati, OH, USA; 3 Department of Pathology, Microbiology and Immunology, Vanderbilt University School of Medicine, Nashville, TN, USA; 4 Department of Pharmacology and Toxicology, University of Alabama at Birmingham, Birmingham, AL, USA; 5 Department of Medicine, University of Washington School of Medicine, Seattle, WA, USA; 6 Department of Medicine, Vanderbilt University School of Medicine, Nashville, TN, USA

**Keywords:** apolipoprotein A-I, ATP binding cassette transporter ABCA1, cholesterol efflux, electron microscopy, high-density lipoprotein, lecithin:cholesterol acyltransferase, multilamellar vesicles, cholate dialysis, spontaneous phospholipid microsolubilization, double belt model, APOA1, apolipoprotein A-I, BHK, baby hamster kidney, Cys, cysteine, DMPC, dimyristoylphosphatidylcholine, EM, electron microscopy, PAGGE, polyacrylamide gradient gel electrophoresis, rHDL, reconstituted HDL

## Abstract

Because of its critical role in HDL formation, significant efforts have been devoted to studying apolipoprotein A-I (APOA1) structural transitions in response to lipid binding. To assess the requirements for the conformational freedom of its termini during HDL particle formation, we generated three dimeric APOA1 molecules with their termini covalently joined in different combinations. The dimeric (d)-APOA1C-N mutant coupled the C-terminus of one APOA1 molecule to the N-terminus of a second with a short alanine linker, whereas the d-APOA1C-C and d-APOA1N-N mutants coupled the C-termini and the N-termini of two APOA1 molecules, respectively, using introduced cysteine residues to form disulfide linkages. We then tested the ability of these constructs to generate reconstituted HDL by detergent-assisted and spontaneous phospholipid microsolubilization methods. Using cholate dialysis, we demonstrate WT and all APOA1 mutants generated reconstituted HDL particles of similar sizes, morphologies, compositions, and abilities to activate lecithin:cholesterol acyltransferase. Unlike WT, however, the mutants were incapable of spontaneously solubilizing short chain phospholipids into discoidal particles. We found lipid-free d-APOA1C-N and d-APOA1N-N retained most of WT APOA1’s ability to promote cholesterol efflux via the ATP binding cassette transporter A1, whereas d-APOA1C-C exhibited impaired cholesterol efflux. Our data support the double belt model for a lipid-bound APOA1 structure in nascent HDL particles and refute other postulated arrangements like the “double super helix.” Furthermore, we conclude the conformational freedom of both the N- and C-termini of APOA1 is important in spontaneous microsolubilization of bulk phospholipid but is not critical for ABCA1-mediated cholesterol efflux.

Apolipoprotein A-I (APOA1), the most abundant protein in HDL, plays an important role in reverse cholesterol transport (1, 2, 3). Human plasma APOA1 exists in both lipid-poor and lipid-bound forms, which differ significantly in conformation (4). In the lipid-poor state, APOA1 can interact with the ABCA1 to promote lipid efflux and formation of nascent HDL particles (5). In the lipid-bound state, APOA1 helical content is increased allowing it to interact with HDL accessory proteins such as LCAT, which catalyzes the formation of cholesteryl esters, thereby increasing cholesterol loading of HDL and formation of larger spherical particles. Recent work has also shown that certain small, lipidated HDLs also promote lipid efflux by the ABCA1 pathway (6, 7). However, the structural plasticity of APOA1 has complicated high-resolution characterizations of the full-length protein, hindering a comprehensive understanding of HDL function (8). Important information about APOA1 structure and function has been obtained from two reported crystal structures of the truncated forms of lipid-free APOA1, each lacking either the N-terminus or the C-terminus (9, 10). The most widely accepted structure of lipid-bound APOA1 is the double-belt model of HDL in which stacked monomers arrange in an antiparallel fashion to encapsulate a circular patch of phospholipid bilayer. This arrangement orients helix 5 in monomer 1 in close proximity with helix 5 in monomer 2 to maximize intermolecular protein interactions (11) and provide stability to the lipoprotein assembly. In addition, all terminal ends of both monomers are proposed to localize to the same region of the particle where they may be in close enough proximity to interact with one another.

Lipid-poor APOA1 acts as an acceptor for cellular lipids via the cell membrane protein ABCA1 thus generating nascent HDL (12, 13, 14). The C-terminus of APOA1, being the most lipophilic portion of the protein, modulates lipid binding. The N-terminal region also displays moderate phospholipid binding ability (15, 16). Mutational studies have demonstrated that truncation of the APOA1 C-terminus (residues 185–243) significantly reduces ABCA1-dependent cholesterol efflux (17, 18, 19, 20). However, a slight reduction in efflux occurs with the N-terminal deletions as well (21). Brubaker *et al.* (22) recently demonstrated that limiting the unfolding of the APOA1 N-terminal bundle via an intramolecular disulfide linkage inhibits its lipidation. These observations fit with the proposal from Palgunachari *et al.* (23) that the N- and C-terminal portions of APOA1 first contact lipid to initiate a zipper like incorporation of the intervening helical domains to complete lipid binding. This synchronization likely requires conformational flexibility at both the termini.

Here, we performed experiments to determine how the tethering of two APOA1 molecules to each other at their termini, in all three possible combinations, affects both the structure of reconstituted HDL (rHDL) particles and the ability of the lipid-free proteins to reorganize lipids to generate HDL particles. The results suggest that the N- and C-termini are indeed in close proximity in lipidated HDL particles, however, they need significant conformational freedom to spontaneously rearrange lipid into discoidal HDL particles.

## Materials and methods

### Preparation of termini-tethered APOA1 dimers

**Fig. 1:**
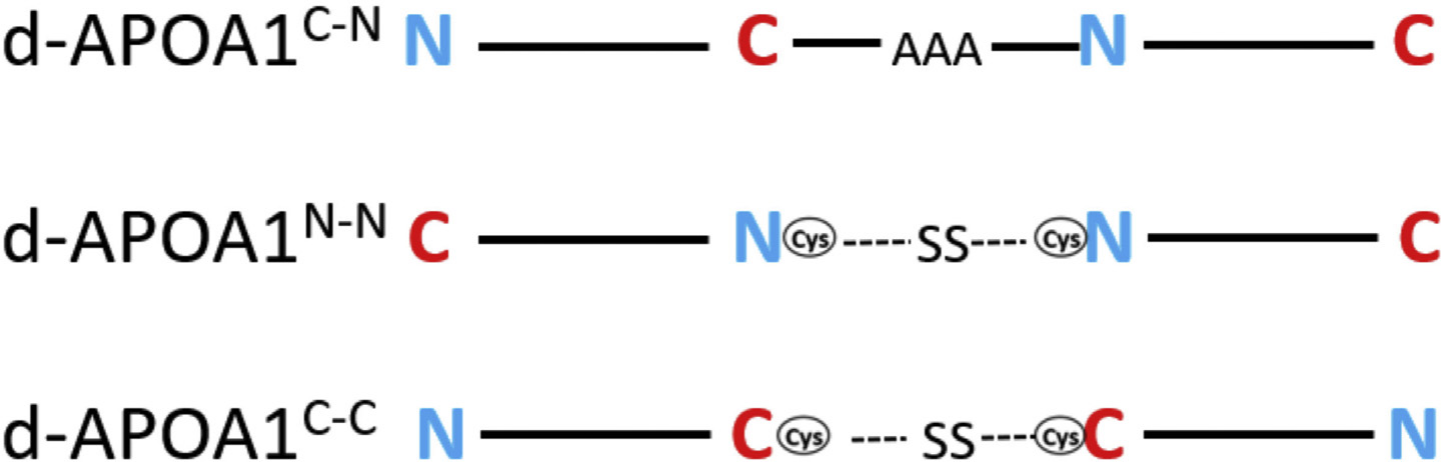
Mutant design for generating covalently connected APOA1 dimers. Fusion construct d-APOA1^C-N^ contains two APOA1 molecules in a head-to-tail arrangement via an alanine linker (AlaAlaAla). d-APOA1^N-N^ and d-APOA1^C-C^ mutants lock the N- and the C-termini of two APOA1 molecules together via a cysteine residue introduced at the N-terminus and C-terminus, respectively. Disulfide bonds are shown as –SS-. APOA1, apolipoprotein A-I.

pET-30 APOA1 (UniProt ID: P02647) (24) plasmid was used as a template to construct mutant plasmids by site-directed mutagenesis. For this study, we covalently connected two APOA1 molecules together at their termini in three combinations (Fig. 1). The first mutant contained two APOA1 molecules connected tail-to-head with a triple alanine linker (d-APOA1^C-N^). This was accomplished by inserting a second copy of the APOA1 coding sequence immediately after the first, terminating with a stop codon. Then, site-directed mutagenesis was used to add three alanines in between the two copies of APOA1. The second mutant, d-APOA1^N-N^, connected two APOA1 molecules at their N-termini. This was accomplished by replacing the N-terminal Gly in our construct with a cysteine (Cys) residue. The construct was expressed in cells, purified by the integrated 6x His tag followed by proteolytic removal of the N-terminal tag as described previously (25). Purified protein was dialyzed against phosphate-buffered saline (pH 7.2) under nonreducing conditions to promote dimer formation. d-APOA1^C-C^ mutants were generated by placing a Cys residue at position 244 in the sequence and dimerizing the expressed protein similarly. To generate covalently linked dimers that could not be freed by cell-secreted reducing agents for the cholesterol efflux assay, intermolecular disulfide linkages were reduced using 5 mM Tris (2-caboxyethyl) phosphine hydrochloride for 1 h at room temperature, followed by removal of Tris (2-caboxyethyl) phosphine hydrochloride using a desalting column (PD-10, Sephadex G-25 resin). The bifunctional maleimide-coupled crosslinking compound BM(PEG)3 (spacer arm 17.8 Å) was added at a 2-fold molar excess, and the mixture was incubated for 1 h at room temperature. The reaction was quenched with 10 mM dithiothreitol and incubated for 30 min at room temperature. Cross-linked dimers were purified by size-exclusion chromatography using four Superdex 200 columns in tandem. The reaction products were analyzed by SDS-polyacrylamide gradient gel electrophoresis (PAGGE), and protein concentrations were determined using the Markwell modified Lowry assay (26).

### Reconstitution of dimeric APOA1 mutants into HDL particles

rHDL particles were generated using POPC and unesterified (free) cholesterol at a POPC: free cholesterol: protein molar ratio of 85:5:1 by cholate dialysis (25). The particles were isolated on a size-exclusion chromatography column (Superdex 200 10/300), and the homogeneity of the preparation was evaluated by native 8%–25% PAGGE (Pharmacia) as described (25). Protein content and phospholipid concentrations in the particles were determined as described (25).

### Negative-stain electron microscopy

Samples were prepared for electron microscopy (EM) using a standard staining protocol (27). Briefly, purified rHDL were pooled and concentrated to a final protein concentration of 0.1 mg/ml. Before staining, the samples were further diluted in a standard Tris buffer to 0.001 mg /ml. Formvar-carbon-coated copper transmission electron microscopy grids were glow discharged in a glow discharge unit (Electron Microscopy Sciences EMS100) at 25 mA current for 2 min at negative polarity. Immediately after glow discharge, a grid was floated on a 20 μl drop of sample for 30 s and excess sample was wicked from the surface. The grid was washed sample-side-down on two 35 μl drops of deionized water, wicked briefly, and then applied to a 40 μl drops of 2% phosphotungstic acid, pH 7.0 for 20 s. Wicking involved touching the edge of filter paper to the grid to remove excess fluid but leaving the grid slightly wet to avoid drying of the sample during staining. The stained grid was then carefully placed in a plastic Petri dish and stored in the dark to air dry. The grids were imaged on a Thermo Fisher Scientific (Philips/FEI) T-12 transmission electron microscope operated at 100 kV. Images were collected at 150,000× magnification (pixel size of 3.9 Å/pixel) with an AMT (Advance Microscopy Techniques) 2K X 2K CCD camera. For quantification, at least 20 arbitrarily selected fields were chosen and more than 250 particles were measured for each condition.

### DMPC binding assay

To evaluate the ability of APOA1 dimers to bind lipids, the efficiency of each protein to clear dimyristoylphosphatidylcholine (DMPC) multilamellar vesicle turbidity was determined. Briefly, 5 mg of DMPC (Avanti Polar Lipids Inc., Alabaster, AL) in chloroform was dried in a borosilicate test tube using nitrogen and placed under vacuum for 1 h. One milliliter of Tris salt buffer was added to a final lipid concentration of 5 mg/ml, and the lipid mixture was sonicated for 20 s. DMPC: protein mass ratio of 2.5:1 in a total volume of 249.8 μl was used to measure lipid binding of each sample in triplicate. Kinetic measurements of lipid clearance were monitored on a spectrophotometer by measuring absorbance at 325 nm as a function of time at 24.5°C.

### Lecithin:cholesterol acyltransferase activity assay

The efficiency of LCAT-catalyzed cholesterol esterification on rHDL was measured according to methods previously described (25). Briefly, tritiated cholesterol was incorporated into rHDL particles containing the various dimeric mutants using sodium cholate dialysis. The particles were incubated with recombinant human LCAT and then, the extent of radioactive cholesteryl ester was determined by quantitative thin layer chromatography and scintillation counting.

### Cholesterol efflux

HDL-mediated cholesterol efflux was measured using mifepristone inducible ABCA-1 expressing baby hamster kidney (BHK) cells (28). BHK cells were cultured in DMEM/high glucose media at 37°C in a humidified 5% CO_2_ atmosphere and radiolabeled by incubation with [H^3^] cholesterol for 16 h, washed twice, and then incubated for 6 h with increasing concentrations of lipid-free APOA1 dimers (2.5–20 μg/ml). Efflux was calculated as the percentage of total [H^3^] cholesterol (medium plus cell-associated) released into the medium of BHK cells stimulated with mifepristone minus that of cells in medium alone. Percent efflux was normalized to the medium alone controls.

## Results

**Fig. 2:**
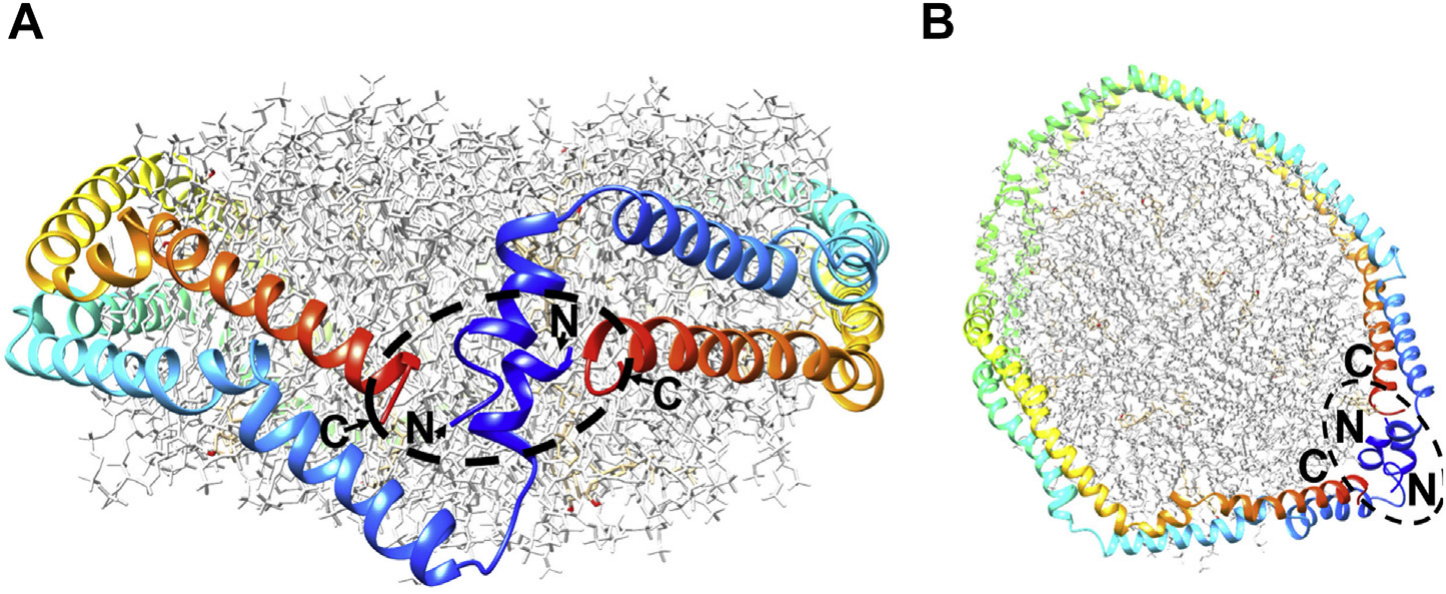
The double belt model for a reconstituted discoidal HDL particle. The amino termini and the carboxyl termini (indicated by the arrows) from both APOA1 monomers localize to the same region (dotted oval region approximately 28 Å in maximal diameter) on the HDL (A) side (B) top view. Apolipoprotein A-I helices are represented as rainbow colored ribbons using UCSF Chimera (figure adapted from ref. (29)). In this model, the N-termini of both APOA1 molecules are predicted to be 17 Å apart, the C-termini are 28.6 Å apart, and the N-C termini of either molecule are 19.7 Å apart. APOA1, apolipoprotein A-I.

The classic double belt model for a discoidal HDL particle predicts that two APOA1 monomers wrap around a patch of phospholipid bilayer in a conformation that positions all four termini in close proximity to one another (11, 29) (Fig. 2). If this model is correct, we hypothesized that tethering the termini of APOA1 molecules in various combinations should not affect the overall size and composition of rHDL particles generated by the cholate dialysis technique. On the other hand, if the termini are not in close proximity in rHDL as predicted by the model, then we expected to see abnormal particles generated with the tethered APOA1 mutants. In our previous work, we developed a consensus model for dimeric full-length APOA1 in its lipid-free state (30). In that structure, the N- and C-termini of molecules A and B, respectively, are in close proximity, within about 30 Å. However, unlike in the discoidal structure, the N- and C-termini of each individual APOA1 molecule are far apart (N-N, 107 Å; N-C, 93 Å). Thus, these tethered mutants also gave us an opportunity to examine the impact of tethering the APOA1 terminals on the process of spontaneous lipid reorganization by lipid-free APOA1.

### Characterization of lipid free APOA1 dimers

**Fig. 3:**
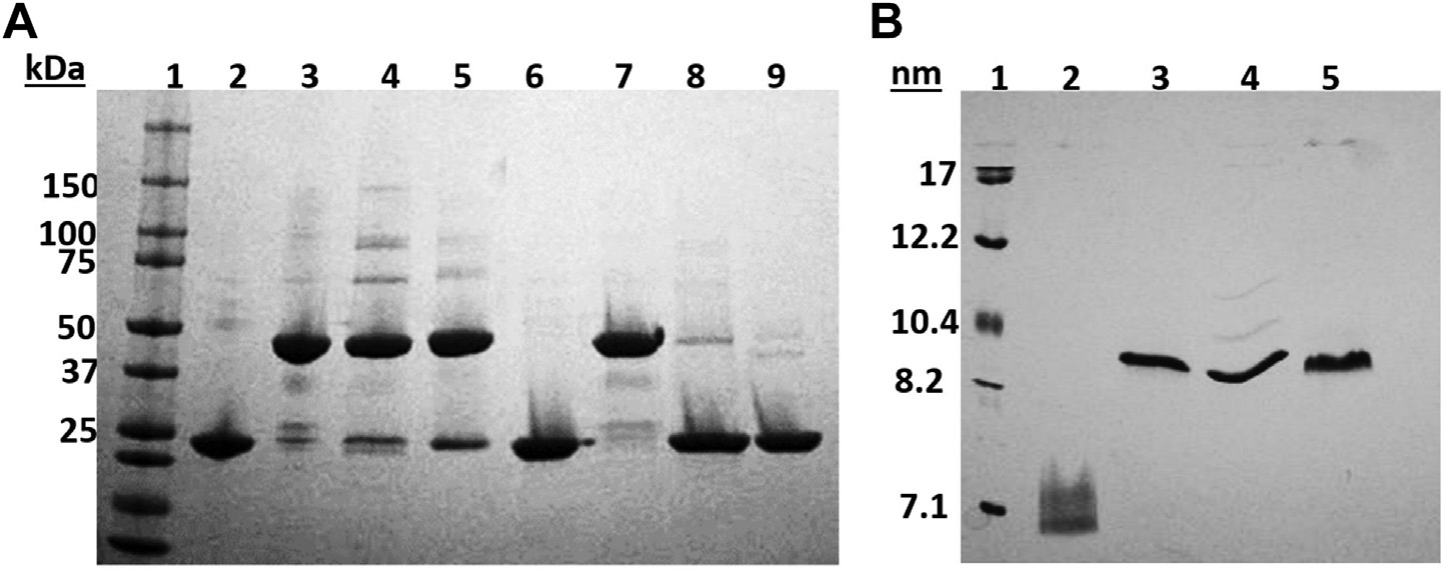
SDS-polyacrylamide and native gel electrophoresis of purified APOA1 proteins. A: Representative SDS PAGGE analysis of recombinant wild-type APOA1 (lanes 2 and 6), d-APOA1 C-N (lanes 3 and 7), d-APOA1 N-N (lanes 4 and 8), and d-APOA1 C-C (lanes 5 and 9) lipid free proteins. The proteins under nonreducing conditions to promote disulfide linkages are shown in lanes 2–5. Proteins reduced with 10 mM DTT to cleave disulfide linkages are shown in lanes 6–9. B: Native PAGGE of proteins loaded in the same order as in (A). The gels were loaded with 4 μg proteins per well and stained with Coomassie Brilliant blue dye. Molecular weight standards are shown in lane 1 of each gel. APOA1, apolipoprotein A-I.

After producing the various covalent dimerized APOA1 species shown in Fig. 1, they were subjected to SDS PAGGE analysis. As expected, WT APOA1 exhibited an apparent size of ∼23 kDa. d-APOA1^C-N^, on the other hand, exhibited an apparent size of about ∼50 kDa indicating the two APOA1 copies were covalently linked. Under nonreducing conditions, the d-APOA1^N-N^ and d-APOA1^C-C^ mutants existed primarily as dimers at an apparent size of ∼50 kDa. The Cys mutant APOA1s tended to have a small amount of monomeric protein (∼5–7%) that likely reflected incomplete formation of disulfide bonds. Under reducing conditions, both Cys mutants (d-APOA1^N-N^ and d-APOA1^C-C^) quantitatively reverted to monomeric bands similar to WT APOA1, showing that the dimerization was entirely because of the formation of disulfide linkages between the introduced Cys residues. Because it contains an alanine linker instead of a disulfide bond, d-APOA1^C-N^ remained dimeric under reducing conditions (Fig. 3A). Native PAGE analysis showed the signature ‘smear’ band for WT APOA1, likely because of its multiple states of oligomerization in solution (Fig. 3B). Interestingly, all of the dimeric APOA1 mutants migrated primarily as a tight band with a hydrodynamic diameter of around 9 nm. We suggest that this occurred because WT APOA1 oligomers, which form in a concentration and equilibrium-based manner, dissociate and reassociate during the native gel run, explaining the smaller size of WT APOA1. The covalent dimers, on the other hand, cannot dissociate during the native gel separation and therefore appear larger.

### Characterization of rHDL particles produced by cholate dialysis

**Fig. 4:**
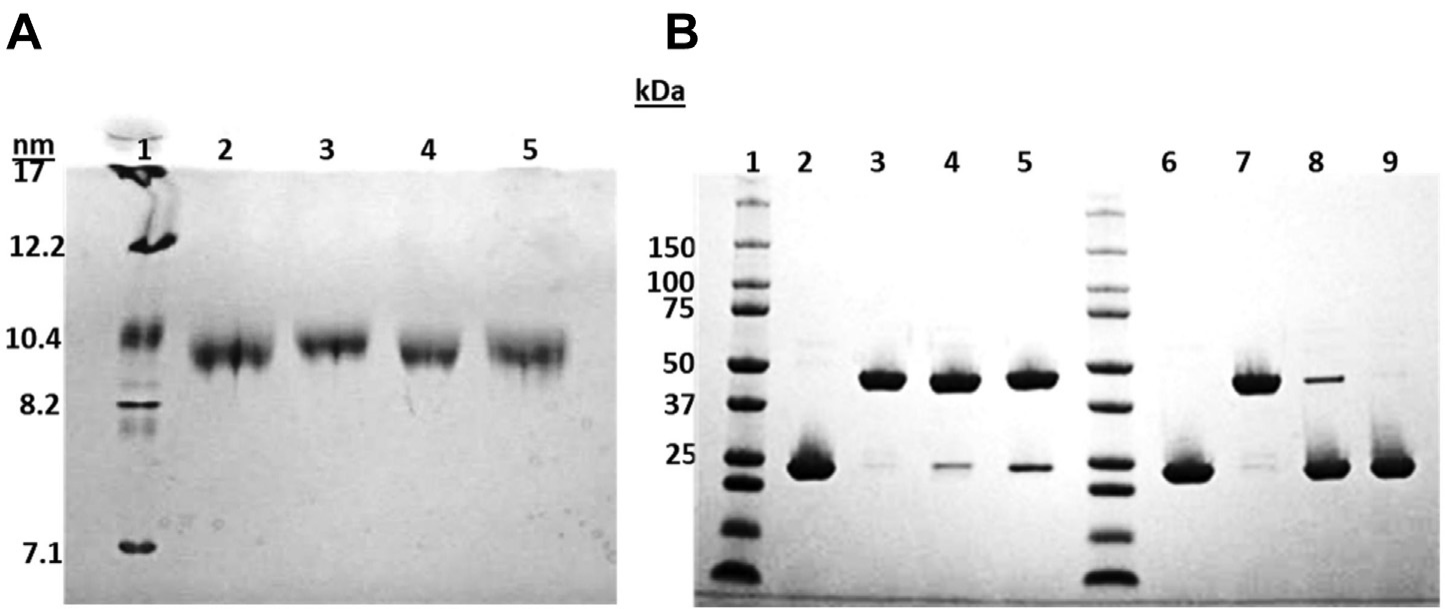
Characterization of rHDL particles generated with each APOA1 mutant. A: Representative native PAGGE analysis of reconstituted HDL particles containing wild-type, d-APOA1^C-N^, d-APOA1^N-N^, and d-APOA1^C-C^ (lanes 2–6, respectively) from three independent replicates. B: SDS-PAGE analysis of samples loaded in the same order as in (A) under nonreducing (lanes 2–5) and reducing (lanes 6–9) conditions. Molecular weight standards are shown in lane 1 of each gel. APOA1, apolipoprotein A-I; rHDL, reconstituted HDL.

We generated reconstituted discoidal HDL particles with each mutant using the classic cholate dialysis method as described in Materials and methods. Figure 4A shows that all proteins readily formed homogeneous rHDL particles that migrated as single monodisperse bands with an apparent size of ∼9–10 nm positions by nondenaturing gradient gel electrophoresis. Figure 4B shows that all the particles generated with the mutants behaved similarly to the corresponding lipid-free versions in Fig. 3 under oxidizing and reducing conditions. All particles were of comparable composition with respect to phospholipid to protein ratios (Table 1). The calculated hydrodynamic diameters by native PAGE were overall similar, though the particles made with d-APOA1^C-N^ appeared slightly larger by this technique. This suggests that tethering APOA1 helices at the termini did not negatively impact its ability to form rHDL particles, at least when lipid solubilization is facilitated by detergents.

**Table 1:** Composition and physical properties of rHDL particles generated with the APOA1 dimeric mutants derived from three independent replicates

Protein Component of rHDL	Start PL:Pro (mol:mol)	Final PL:Pro (mol:mol)	Diameter (nm) nPAGGE^a^	Diameter (nm) EM^b^
WT-APOA1	80:1	81 ± 10:1	9.3 ± 0.1	10.8 ± 2
d-APOA1^C-N^	80:1	79 ± 16:1	9.7 ± 0.1	11.8 ± 2
d-APOA1^N-N^	80:1	83 ± 19:1	9.3 ± 0.1	11.9 ± 2
d-APOA1^C-C^	80:1	74 ± 19:1	9.3 ± 0.1	11.5 ± 2

EM, Electron microscopy; NPAGGE, Nondenaturing polyacrylamide gradient gel electrophoresis.

a Determined on an 8–25% native Phast gel under native conditions using high molecular weight protein standards (GE Healthcare, Amersham).

b Determined from the particle size distribution histograms with Gaussian function fit (in curve) using GraphPad Prism version 9.0.0. All data show mean ± 1 standard deviation.

**Fig. 5:**
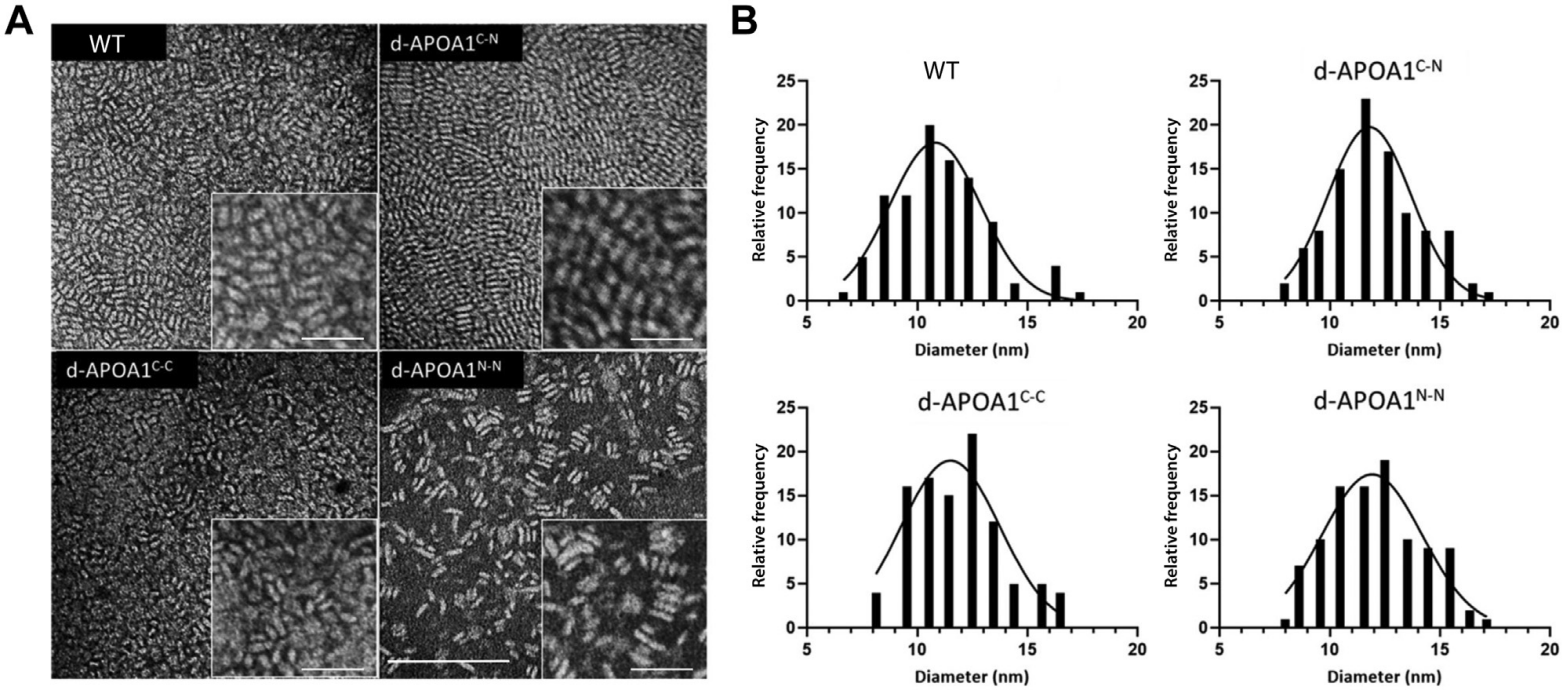
Size and morphology of rHDL particles generated with dimeric APOA1 mutants measured by negative stain electron microscopy. A: Negative stain electron micrographs of APOA1-/POPC-containing reconstituted HDL particles. Insets: zoomed-in views illustrate stacking rouleau in the samples. The bar in the unzoomed image represents 100 nm. The bars in the zoomed images for WT and d-APOA1C-N represent 25 nm. The bars in the zoomed images for d-APOA1C-C and d-APOA1N-N represent 19 nm. B: Particle size distribution (histogram) with Gaussian function fit (in curve) displaying the frequency of the distribution of diameters of particles shown in (A). Similar results were obtained from two independent preparations of rHDL particles. APOA1, apolipoprotein A-I; rHDL, reconstituted HDL.

To examine the morphology and structure of the HDL particles in more detail, samples were prepared for negative stain EM. The EM micrographs showed that particles stacked together into rouleau and were discoidal in shape (Fig. 5A). The distribution of the quantified particle diameters particles was similar among the WT and the mutants with peak diameters of about 11–12 nm (Fig. 5B and Table 1). By this sizing technique, the WT APOA1-containing particles trended slightly smaller than those produced with the mutants, but statistically all rHDL preparations were of similar size. In our experience with negative staining of discoidal lipoproteins, diameter measurements are commonly slightly larger than those calculated by PAGGE. The reason may have to do with lack of wetting by stain solution at the lipoprotein surface. Overall, the data indicate that coupling the APOA1 termini in various combinations had little impact on the size, composition, and or morphology of rHDL particles produced by cholate dialysis.

### Lipid binding characteristics of mutant APOA1 proteins

**Fig. 6:**
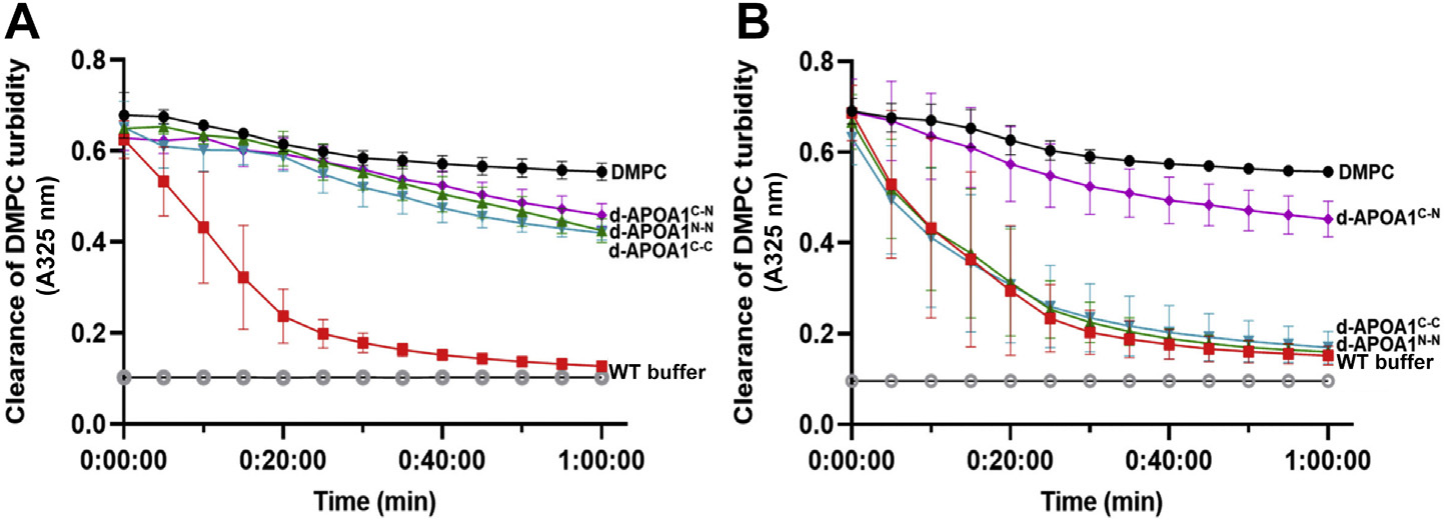
APOA1 dimeric mutants binding to DMPC liposomes. Changes in turbidity of DMPC vesicles was monitored by the change in absorbance at 325 nm at 30 s intervals for 60 min and plotted as a function of time in (A) oxidized or (B) reduced states. All traces represent means with error bars representing the standard deviation (SD) of measurements performed in triplicates. APOA1, apolipoprotein A-I.

Lipid-free APOA1 spontaneously solubilizes DMPC multilamellar vesicles to form discoidal HDL particles when incubated at the gel to liquid crystalline transition temperature of the lipid (31, 32). APOA1 is thought to insert its hydrophobic helices into lipid packing defects on the surface of these multilamellar liposomes and then ‘microsolubilize’ a disc-sized patch of the bilayer by an as yet ill-defined mechanism. We measured the kinetics of DMPC turbidity clearance of our mutant APOA1 proteins versus WT APOA1. The lipid-free proteins were mixed with DMPC suspensions at a mass ratio of 2.5:1 phospholipid: protein, and the turbidity of DMPC multilamellar vesicles was measured as a function of time. Figure 6 shows that addition of WT APOA1 caused a rapid clearance of DMPC turbidity with the reaction mostly complete by 20 min. By contrast, equal masses of all three dimeric APOA1 mutants largely failed to clear DMPC, performing only marginally better than adding vehicle buffer to the liposomes. However, when the dimers were reduced, d-APOA1^N-N^ and d-APOA1^C-C^ performed similarly to WT APOA1. No change in DMPC clearance was observed with the alanine linked d-APOA1^C-N^ in the reduced state (Fig. 6B). These results demonstrate that tethering the APOA1 termini profoundly impacts the ability of APOA1 to spontaneously reorganize lipids and suggests that the termini likely need to move apart during this reaction and/or that a decrease in the mobility of the dimers impairs lipid reorganization.

### LCAT activity

**Fig. 7:**
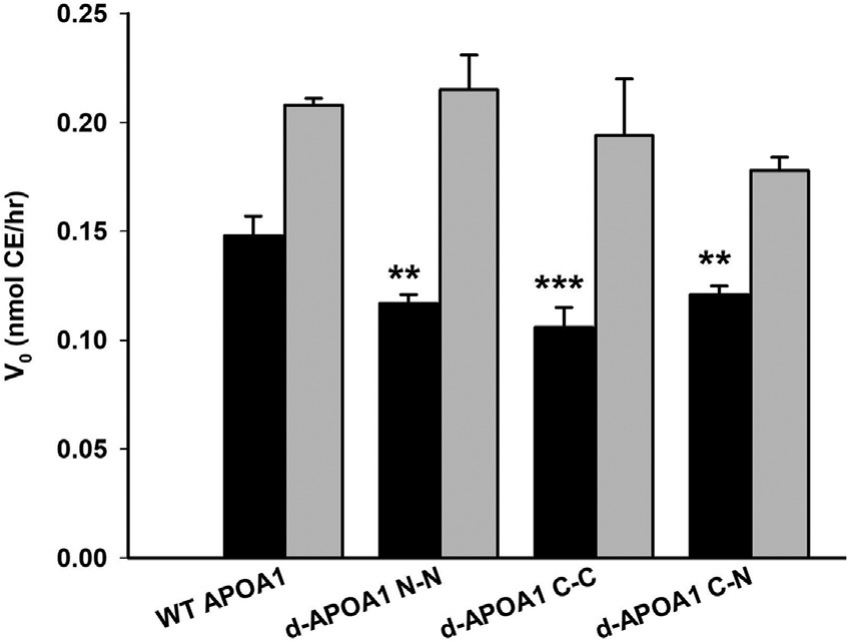
Lecithin:cholesterol acyltransferase activation by rHDL particles. Particles were generated with [^3^H] cholesterol and incubated with LCAT in the oxidized (solid) or reduced (gray) states. The amount of labeled CE, as determined by thin-layer chromatography, was quantitated by scintillation counting. The bars show mean ± SDs of triplicate measurements for each condition. One-way analysis of variance: ∗*P* < 0.05, ∗∗*P* < 0.01, ∗∗∗*P* < 0.001) using SigmaPlot (v.11.0) was used to determine differences in the reaction velocity (V_0_) of CE production between wild-type and mutant APOA1 rHDL. APOA1, apolipoprotein A-I; rHDL, reconstituted HDL.

We studied the ability of all rHDL (made by cholate dialysis) to act as substrates for cholesterol esterification by LCAT. WT APOA1 particles were efficient substrates for LCAT (Fig. 7) as expected. Under oxidizing conditions, there was a modest statistically significant decrease in LCAT activity for all of the termini-coupled mutants relative to WT APOA1. However, all particles retained about 80% of WT LCAT activity and thus were still reasonably effective activators of the enzyme. Under reducing conditions, where the Cys-linked dimers were freed to monomers, all the mutants displayed LCAT activity that was comparable to WT. Increased LCAT activity in the reduced state with Cys lacking WT and d-APOA1^C-N^ is consistent with our previously published result that beta-mercaptoethanol enhances LCAT activity (25).

### Cholesterol efflux capacity

**Fig. 8:**
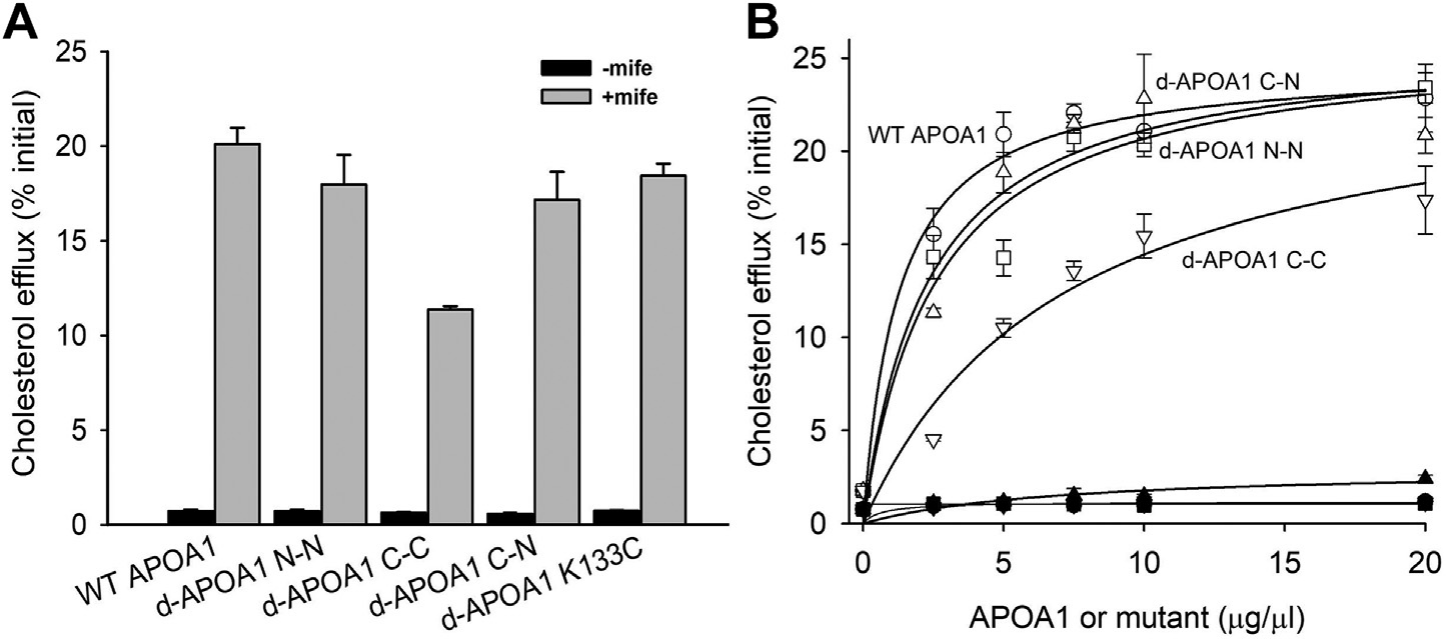
Effect of covalently modifying termini of lipid-free APOA1 and dimeric mutants on cholesterol efflux. A: Cholesterol efflux to APOA1 (10 μg/ml) was measured in BHK cells treated with (gray) or without (black) mifepristone to induce expression of human ABCA1. The data are mean ± 1 sample SD of triplicate wells from three independent experiments. One-way analysis of variance: (∗*P* < 0.05, ∗∗*P* < 0.01, ∗∗∗*P* < 0.001) using SigmaPlot (v.11.0) was used to determine differences between wild-type and mutant APOA1. B: Concentration dependence of each lipid-free mutant on ABCA1-mediated cholesterol efflux. Nonlinear regression fits to the data for each mutant represent one site ligand binding curves as fit by SigmaPlot version 11.2 (f = Bmax∗x)/(Kd + x), where Bmax represents maximal efflux independent of acceptor concentration and Kd represents the half maximal concentration of acceptor. APOA1, apolipoprotein A-I; BHK, baby hamster kidney.

The ability of coupled APOA1 dimers in the lipid-free form to promote cholesterol efflux via ABCA1 was tested in BHK cells that can toggle ABCA1 expression on or off using a mifepristone-inducible promoter (28). To eliminate the possibility that linked APOA1 dimers might revert to monomeric forms in the reducing environment of cells, we generated cross-linked APOA1 dimers using an irreversible sulfhydryl specific crosslinker as described in Materials and methods. Mutants joined in this way remained dimeric even under aggressively reducing conditions of 3 μM beta-mercaptoethanol (not shown). Figure 8A shows that cholesterol efflux was low in the absence of mifepristone (no ABCA1 expression, black bars), indicating a low contribution of cholesterol efflux from non-ABCA1-mediated pathways. Upregulation of ABCA1 resulted in a robust increase in cholesterol efflux to 10 μg/ml of all APOA1 variants tested (Fig. 8A). Wild-type, d-APOA1^N-N^, and d-APOA1(K133C) (included as an example of a dimeric APOA1 mutant that was not tethered at one of its termini) displayed comparable ABCA1-mediated cholesterol efflux activity. The alanine-linked d-APOA1^C-N^ also displayed WT-like efflux efficiency though slightly lower than WT in this experiment. However, the activity of d-APOA1^C-C^ mutant was decreased by ∼50% compared to WT APOA1 as determined by a one-way ANOVA. To gain further information about the concentration dependence of this effect, we performed a repeat experiment where the concentration of each APOA1 variant was increased from 0 to 20 μg/ml (Fig. 8B). Again, WT APOA1, d-APOA1^N-N^, and d-APOA1^C-N^ showed similar concentration dependence achieving an apparent maximal velocity of around 25% of initial cell cholesterol at 20 μg/ml with half maximal concentrations of 1–3 μg/ml. d-APOA1^C-C^ promoted reduced cholesterol efflux at all concentrations tested. Nonlinear regression using a one-site saturation binding model suggested that d-APOA1^C-C^ would reach a similar Vmax of ∼25% as the other mutants, but its half maximal concentration was 7 μg/ml, 2–7 times higher than the other variants.

## Discussion

A previous study by Ohnsorg (33) showed that a trimeric form of APOA1, presumably associated at the N-terminus, produced differently sized rHDL particles versus WT APOA1 that were partially impaired in activating LCAT. But the lipid-free trimer promoted ABCA1-mediated cholesterol efflux comparably to WT APOA1. In this study, we explored the functionality of covalent dimers of APOA1 tethered at different combinations of their termini. The major findings were as follows, *i*) N-N, C-N, and C-C tethered APOA1 dimers all formed rHDL discs with similar composition, diameter, and shape as unlinked WT APOA1 when assisted by cholate dialysis, *ii*) however, all dimers were near completely deficient at spontaneously reorganizing phospholipid from liposomes to discs in the absence of detergent assistance, *iii*) despite this, the N-N and C-N dimers were able to promote ABCA1-mediated cholesterol efflux comparable to unlinked WT APOA1. These observations provide clues to the mechanism of HDL assembly and are discussed in turn below.

**Fig. 9:**
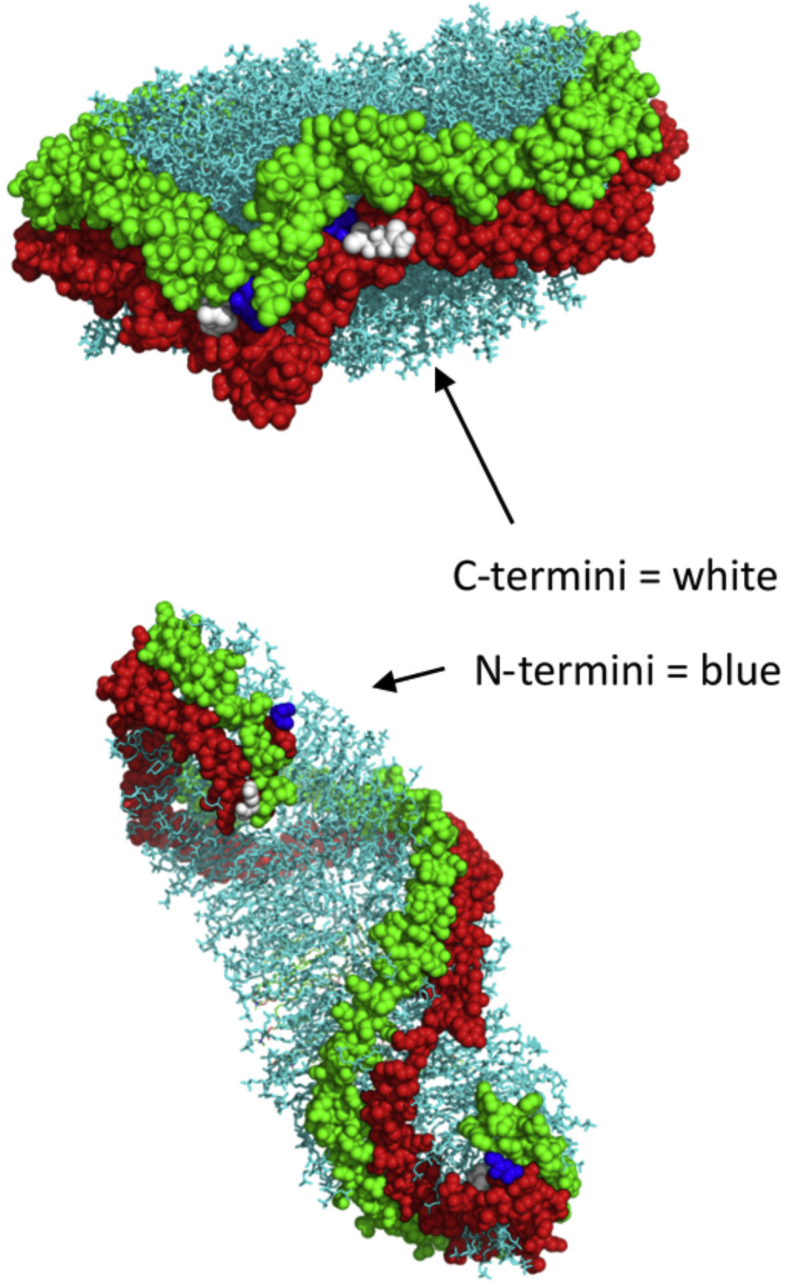
Comparison of APOA1 termini position in the double belt model (top) and double superhelix (bottom). Each of two APOA1 molecules within a rHDL particle are colored in green and red. APOA1, apolipoprotein A-I; rHDL, reconstituted HDL.

As shown in Fig. 2, Fig. 9, the double belt model of two APOA1 molecules in a discoidal particle places the N- and C-termini of both copies of APOA1 in close proximity on the disc edge. Our results clearly show that, irrespective of the tethering, all mutants could be induced to produce discoidal rHDL that look compositionally and morphologically like those produced by WT APOA1. These particles were functional as they activated LCAT with reasonably comparable effectiveness. Our previous work showed that the registry, that is, the rotation of the APOA1 rings with respect to each other on the disc edge, is critically important for LCAT activation (25). Therefore, the registry and overall structure of all the tethered dimer mutants seems similar to particles generated with WT APOA1. This observation strongly supports the double belt model as well as its ‘belt and buckle’ and ‘solar flares’ variations (34, 35, 36). However, the fact that the N-N and the C-C dimers generate normal rHDL discs strongly argues against the ‘double super helix’ model proposed by Wu *et al.* (37) based on small angle neutron scattering, echoing previous challenges to this model (38). In this extended, rod-like model (Fig. 9) (Table 2), the C-termini of monomers A and B are predicted to be on opposite sides of the particle, some 92 Å apart, as are the N-termini of each (107 Å apart). Tethers at the N-N and C-C would dramatically disrupt the morphology and, likely, the composition of particles that adopted this conformation. The only explanation (at least that we can think of) for the ability of the tethered APOA1 molecules to produce normal particles is if they are arranged in a circular belt with all termini meeting up on the disc edge.

**Table 2:** Predicted distances between the amino and carboxyl termini of two APOA1 monomers the double belt and double superhelix models APOA1 in an rHDL particle

APOA1 Monomer	APOA1 Monomer	Double Belt Model Measurements (Å)^a^	Double Super Helix Model Measurements (Å)
A	A		
N-terminus	C-terminus	6.3	92.0
B	B		
N-terminus	C-terminus	6.3	103.6
A	B		
N-terminus	N-terminus	14.5	107.7
N-terminus	C-terminus	15.8	16.4
C-terminus	C-terminus	25.4	92.2

a Models obtained from ref. (29) and (34). Distance measurements were obtained using PyMOL.

All mutants could generate normal rHDL when assisted by cholate solubilization of the phospholipid substrate (i.e., the cholate dialysis method), but the lipid-free mutants could not spontaneously generate those rHDL by microsolubilizing DMPC at its gel/liquid-crystal phase transition temperature. However, once the Cys-linked dimers were released under reducing conditions, the mutants regained WT ability to solubilize the lipids. This indicates that the Cys mutations were not responsible for the effect, only the disulfide linkages themselves. APOA1 is thought to use its C- and N-terminal helical domains to exploit packing defects in the partially melted lipids to *i*) bind to and then *ii*) reorganize the phospholipids into discoidal patches of bilayer. The fact that these mutants could not do this suggests two possibilities. First, the termini may need to be able to move apart from each other at some point during binding or reorganization of the lipid. In other words, the tethers prevented APOA1 from adopting conformational changes required to solubilize the lipids during the reaction. Second, the loss of either the N- or C-terminus, through tethering, prevents APOA1 from binding lipid packing defects. Palgunachari *et al.* (23) proposed that the N- and C-termini make first contact with a lipid surface. These form anchor points that direct the intervening helical domains in APOA1 to cooperatively bind lipid and solubilize it. Limiting the freedom of either of these may eliminate the ability of the molecule to gain a ‘foothold’ on the lipid to initiate lipid solubilization. During the cholate dialysis procedure, the detergent does the ‘work’ of solubilizing the lipid into micelles before APOA1 addition. The fact that the mutants could produce rHDL particles effectively by cholate dialysis, but not by spontaneous lipid reorganization, suggests that conformational freedom of both APOA1 termini is critical for the initial step of attacking an intact lipid bilayer, but is not important once the bilayer structure has been broken down by another means.

Another intriguing finding is that the lipid-free dimers, for the most part, were quite capable of promoting cholesterol efflux via ABCA1. The observation that the C-C tethered mutant was defective at all concentrations tested and showed a much higher half-maximal efflux concentration is not surprising as many studies have shown that the C-terminal helix of APOA1 is critically important for this mechanism, thus limiting its conformational freedom is consistent with the reduced cholesterol efflux we observed (39, 40, 41). The fact that the N-N and the C-N were comparable to WT APOA1 across all concentrations tested indicates that they can effectively perform all steps required for this process with similar efficiency. This clearly indicates that the mechanism of ABCA1 cholesterol efflux differs from the spontaneous lipid solubilization that occurs in the DMPC clearance assay. Phillips (42) have suggested that ABCA1 is a transmembrane phospholipid floppase that moves lipids from the inner leaflet of the plasma membrane to the outer leaflet. They proposed that this causes localized packing defects in nearby regions of the plasma membrane that allows APOA1 to bind to and solubilize a patch of membrane, leaving as a nascent HDL particle. By contrast, Ueda and Segrest have proposed ‘direct binding’ models whereby APOA1 makes specific contacts with ABCA1 (43, 44) to extract lipid that is accumulated in the extracellular domains of the transporter. Although not absolutely conclusive, the discordance between the performance of the dimeric mutants in the DMPC clearance assay versus the ABCA1 lipid efflux assays argues against a straight microsolubilization mechanism being responsible for ABCA1-mediated lipid efflux. It also suggests that at least the N termini can remain in close proximity throughout transition from lipid-free to lipid-bound nascent HDL particle during the process of ABCA1-mediated lipid efflux. It is also worth noting that, on a mass basis, dimeric APOA1 is about as efficient as monomeric APOA1 for ABCA1-mediated lipid efflux. Ueda has suggested that APOA1 must dimerize to interact with ABCA1 to generate HDL. Although this may indeed be the case, it is clear that pushing the equilibrium to nearly 100% APOA1 dimer before the ABCA1 reaction does not offer any increases in efficiency to the reaction.

In summary, we have demonstrated that the arrangements of APOA1 termini within rHDL are best explained by the double belt model of HDL. In addition, flexibility or conformational freedom of APOA1 termini plays an important role in the spontaneous binding and reorganization of phospholipid but less of a role in ABCA1-mediated lipid efflux. Further work with these stabilized mutants may help us understand how APOA1 conformational flexibility impacts other HDL functions, including its ability to mediate the interactions of some 250 minor HDL proteins which can have profound functional implications in a wide variety of physiological processes.

## Data availability

All data is available upon request to the corresponding author.

## Conflict of interest

The authors state no conflict of interest.
